# Survival outcomes of appendiceal mucinous neoplasms by histological type and stage: Analysis of 266 cases in a multicenter collaborative retrospective clinical study

**DOI:** 10.1002/ags3.12241

**Published:** 2019-02-25

**Authors:** Toshinori Sueda, Kohei Murata, Takashi Takeda, Yoshinori Kagawa, Junichi Hasegawa, Takamichi Komori, Shingo Noura, Kimimasa Ikeda, Masaki Tsujie, Masayuki Ohue, Hirofumi Ota, Masakazu Ikenaga, Taishi Hata, Chu Matsuda, Tsunekazu Mizushima, Hirofumi Yamamoto, Mitsugu Sekimoto, Riichiro Nezu, Masaki Mori, Yuichiro Doki

**Affiliations:** ^1^ Colorectal Group Clinical Study Group of Osaka University (CSGO) Osaka Japan; ^2^ Department of Surgery Osaka Rosai Hospital Sakai Japan; ^3^ Department of Surgery Kansai Rosai Hospital Amagasaki Japan; ^4^ Department of Gastroenterological Surgery Osaka University Graduate School of Medicine Suita Japan; ^5^ Department of Surgery Osaka General Medical Center Osaka Japan; ^6^ Department of Surgery Toyonaka Municipal Hospital Toyonaka Japan; ^7^ Department of Surgery Minoh City Hospital Minoh Japan; ^8^ Department of Surgery Sakai City Medical Center Sakai Japan; ^9^ Department of Gastroenterological Surgery Osaka International Cancer Institute Osaka Japan; ^10^ Department of Surgery Ikeda City Hospital Ikeda Japan; ^11^ Department of Gastroenterological Surgery Higashiosaka City Medical Center Higashiosaka Japan; ^12^ Department of Therapeutics for Inflammatory Bowel Diseases Graduate School of Medicine Osaka University Suita Japan; ^13^ Division of Health Sciences Graduate School of Medicine Osaka University Suita Japan; ^14^ Department of Surgery National Hospital Organization Osaka National Hospital Osaka Japan; ^15^ Department of Surgery Nishinomiya Municipal Central Hospital Nishinomiya Japan; ^16^ Department of Surgery and Science Graduate School of Medical Sciences Kyushu University Fukuoka Japan

**Keywords:** appendiceal carcinoma, mucinous, non‐mucinous, prognosis, survival outcomes

## Abstract

**Aim:**

Appendiceal mucinous neoplasms are rare, and thus the literature is sparse with regard to histological types, staging, and prognosis. In particular, it is unclear how long‐term outcome may differ between mucinous adenocarcinomas and other adenocarcinomas. In the present study, we aimed to investigate the histological types and stages of appendiceal neoplasms, and to evaluate the prognostic impacts of these factors in patients with mucinous adenocarcinomas and non‐mucinous adenocarcinomas.

**Methods:**

Patients with appendiceal tumors diagnosed between 2007 and 2016 were retrospectively identified from the databases of 19 institutions in the Clinical Study Group of Osaka University, Colorectal Group.

**Results:**

A total of 266 patients with appendiceal tumors were identified, of whom 130 had pathologically diagnosed adenocarcinomas, including 57 with mucinous adenocarcinomas and 73 with non‐mucinous adenocarcinomas. Five‐year overall survival (OS) rates were 64.5% for mucinous adenocarcinomas, and 49.0% for non‐mucinous adenocarcinomas. OS was significantly shorter among patients with non‐mucinous adenocarcinomas compared to mucinous adenocarcinomas. Among patients with mucinous adenocarcinomas, 5‐year OS rates were 53.6% for stage 0/I, 82.6% for II/III, and 48.4% for IV. Among patients with non‐mucinous adenocarcinomas, 5‐year OS rates were 90.9% for stage 0/I, 68.8% for II/III, and 7.1% for IV. Analysis of patients with stage IV disease revealed significantly shorter OS among patients with non‐mucinous adenocarcinomas compared to mucinous adenocarcinomas.

**Conclusion:**

Our present findings showed a better prognosis in patients with mucinous adenocarcinomas compared to non‐mucinous adenocarcinomas. In this setting, Union for International Cancer Control staging was associated with prognosis for non‐mucinous adenocarcinomas, but not for mucinous adenocarcinomas.

## 1 INTRODUCTION

The vermiform appendix is the primary site of several distinctive benign and malignant neoplasms. Appendiceal tumors are rare neoplasms, comprising approximately 1% of appendectomy specimens.^1^ The major categories of primary appendiceal neoplasms include epithelial tumors (subclassified as mucinous tumors, neuroendocrine tumors, and mixed glandular and endocrine tumors^2^), mesenchymal tumors, and lymphomas. Mucinous adenocarcinomas are the most common non‐carcinoid tumors of the appendix.^3^ Mucinous neoplasms of the appendix constitute a heterogeneous group of neoplasms, ranging from adenomas to mucinous adenocarcinomas.^4^

Various systems for classifying appendiceal mucinous neoplasms have been proposed by various authors^5^, ^6^, ^7^, ^8^ and in the World Health Organization (WHO) 2010 guidelines.^9^ In an effort to simplify the diagnostic terminology for appendiceal mucinous neoplasms, the WHO has identified morphologic characteristics that can be used to classify low‐grade and high‐grade tumors.^9^ Although our understanding of appendiceal mucinous neoplasms has advanced, their classification remains confusing.

A recent review provides an updated clarification of the various classification systems.^10^, ^11^ The Peritoneal Surface Oncology Group International (PSOGI) has published their consensus regarding the classification and proposed diagnostic terminology for primary appendiceal mucinous neoplasms, which provides rigorous diagnostic criteria for low‐grade appendiceal mucinous neoplasms (LAMNs).^10^ They recommend using the 2016 Modified Delphi Consensus Protocol to classify non‐carcinoid epithelial appendiceal tumors into eight histomorphological architectural groups: adenoma, serrated polyp, LAMN, high‐grade appendiceal mucinous neoplasm (HAMN), mucinous adenocarcinoma (well/moderately/poorly differentiated), signet ring cell low‐differentiated (mucinous) adenocarcinoma, signet‐ring cell (mucinous) adenocarcinoma, and adenocarcinoma. On the other hand, The American Joint Committee on Cancer (AJCC) 8th edition clarifies LAMN staging to include prognostically relevant criteria, and describes a new T category specifically for LAMN, termed Tis(LAMN).^12^ Both the AJCC 8th edition and the PSOGI consensus emphasize the importance of distinguishing between low‐grade and high‐grade intraperitoneal disease, and both advocate for three‐tier grade assessment of appendiceal mucinous neoplasms, in which low‐grade tumors are classified as G1, while high‐grade tumors are classified as G2 or G3.^12^

Appendiceal adenocarcinoma is defined by the presence of infiltrative invasion,^10^ and can be subdivided into mucinous, non‐mucinous, and signet‐ring cell histological types.^13^ Appendiceal adenocarcinoma seems to have different characteristics from other colorectal cancers, but sufficient evidence is lacking due to its rarity. However, the AJCC 8th edition now includes separate classifications for appendiceal carcinomas and colorectal carcinomas.^12^ To date, little is known about variations in long‐term outcomes, but it is expected that long‐term postoperative performance differs between mucinous adenocarcinoma and other adenocarcinomas.

In the present multicenter retrospective clinical study of appendiceal tumors, we aimed to investigate the histological types and stages of appendiceal neoplasms. We further evaluated the prognostic impacts of these factors in patients with mucinous adenocarcinoma and non‐mucinous adenocarcinomas of the appendix.

## 2 PATIENTS AND METHODS

### 2.1 Study design

We retrospectively analyzed a cohort of patients who had been histologically diagnosed with appendiceal tumors between January 2007 and December 2016. This study was approved by our Institutional Review Board (approval number 17019).

### 2.2 Data source and study population

**Figure 1:**
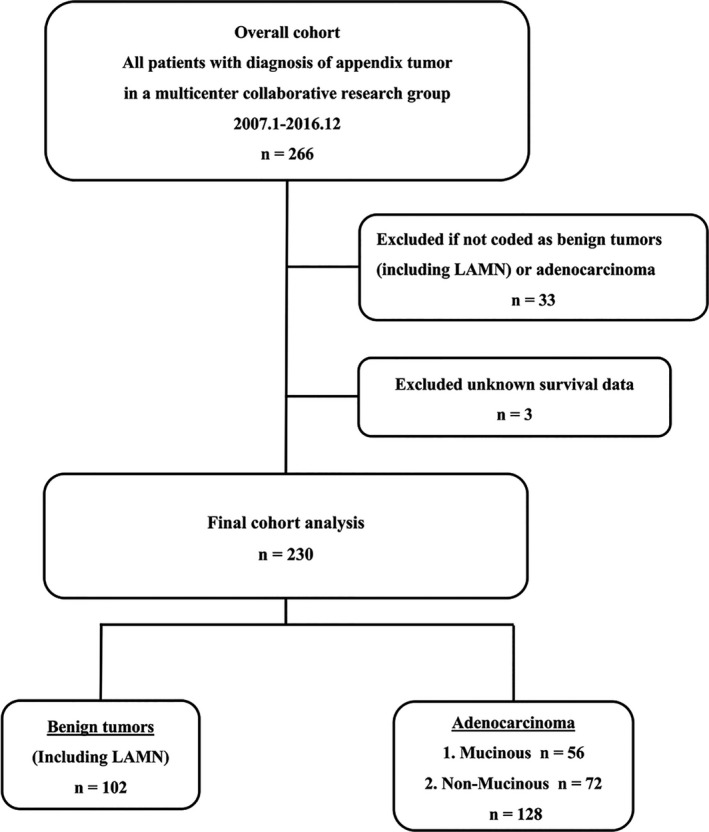
Study flow chart

Data for this study were acquired from the medical records of 19 institutions participating in a multicenter collaborative research group (Clinical Study Group of Osaka University, Colorectal Group). Patients with unknown survival data, and those not coded as benign tumors (including LAMN) or adenocarcinoma, were retrospectively excluded from further analysis (Figure 1).

All recorded clinical and pathological data were validated against medical and pathology records. Recorded variables included age, sex, surgery, surgical stages (one‐stage/two‐stage), surgical approach, surgical procedure, lymph node dissection, combined resection, histological grade, and TNM stage according to the Union for International Cancer Control (UICC) 8th version.^14^ The types of surgical procedure included appendectomy, cecal resection, ileocecal resection (ICR), right hemicolectomy, others, and unknown. The types of operations included one‐stage, two‐stage, others, and unknown. Appendiceal tumors were divided into three histological groups: benign tumors (including LAMN), mucinous adenocarcinoma (well, moderately, poorly, and unknown), and non‐mucinous adenocarcinomas (papillary adenocarcinoma, tubular adenocarcinoma, poorly differentiated adenocarcinoma, and signet‐ring cell carcinoma).

### 2.3 Endpoints

The study endpoint was overall survival (OS), defined as the time in months from the date of surgery to the date of death from any cause. Recurrent disease was diagnosed based on clinical and pathological findings, laboratory results, and diagnostic imaging.

### 2.4 Statistical analysis

We performed descriptive data analyses, calculating frequencies and percentages for categorical variables, and expressing continuous variables as median (range). We evaluated the significance of between‐group differences using the Mann–Whitney *U* and Kruskal–Wallis tests. The Kaplan–Meier method was used to determine the effects of each variable on survival, and log‐rank tests were used to compare survival curves. Hazard ratios (HR) were reported as point estimates with a 95% confidence interval (CI). All statistical analyses were performed using JMP Pro Version 12 (SAS Institute, Inc., Cary, NC, USA). A *P*‐value < 0.05 was considered to indicate statistical significance.

## 3 RESULTS

### 3.1 Baseline characteristics

Figure 1 presents an overview of this study. From the databases of the 19 institutions participating in this study, we identified 266 patients who were diagnosed with appendiceal tumors between January 2007 and December 2016. Table 1 presents the histological characteristics of the overall cohort. Of the 266 patients, 130 (48.9%) were pathologically diagnosed with adenocarcinoma, comprising 57 (43.8%) with mucinous adenocarcinoma, and 73 (56.2%) with non‐mucinous adenocarcinomas. Among the cases of mucinous adenocarcinoma, 18 (31.6%) were subdivided according to histological grade (degree of malignancy), while differentiation grade was unknown for the remaining 39 (68.4%). Of the non‐mucinous adenocarcinomas, 48 (65.7%) were tubular adenocarcinoma, 14 (19.2%) were poorly differentiated adenocarcinoma, five (6.8%) were signet‐ring cell carcinoma, and one (1.4%) was papillary adenocarcinoma. Of the 266 identified patients, 36 were excluded. Thus, the analyzed sample included 230 patients. The distribution by histological type was 102 (44.3%) benign tumors, 56 (24.3%) mucinous adenocarcinomas, and 72 (31.3%) non‐mucinous adenocarcinomas (Figure 1).

**Table 1:** Histological characteristics of the overall cohort (n = 266)

Variables	n				%
Benign tumors (including LAMN)	103				38.7
Malignant epithelial neoplasia	131				49.2
Adenocarcinoma		130			48.8
Mucinous adenocarcinoma (muc)			57		
Well differentiated				6	
Moderately differentiated				9	
Poorly differentiated				3	
Unknown				39	
Papillary adenocarcinoma (pap)			1		
Tubular adenocarcinoma (tub)			48		
Well differentiated (tub1)				25	
Moderately differentiated (tub2)				23	
Poorly differentiated adenocarcinoma (por)			14		
Signet‐ring cell carcinoma (sig)			5		
Unknown			5		
Unknown		1			0.4
Endocrine cell tumor	23				8.6
Carcinoid tumor		2			
Endocrine cell carcinoma		9			
Goblet cell carcinoid		12			
Non‐epithelial tumor	0				0
Lymphoma	2				0.8
Metastatic tumor	2				0.8
Others	5				1.9

LAMN, low‐grade appendiceal mucinous neoplasm.

Table 2 shows the patient baseline characteristics by histological type. The cohort included 93 men (40.4%) and 135 women (58.7%), and the median age was 78 years (range: 18‐94 years). The median age at diagnosis was higher among patients with non‐mucinous adenocarcinomas compared to those with benign tumors and mucinous adenocarcinoma. Regarding operation type, 190 patients (82.6%) underwent one‐stage surgery, whereas 25 (10.9%) underwent two‐stage surgery. Lymph node dissection was performed with 158 (68.7%) of 230 patients, combined resection in 57 patients (24.8%), and R0 resection in 177 patients (76.9%). Patients with benign tumors had higher rates of one‐stage surgery, laparoscopic approach, appendectomy, and R0 resection than the other groups. The group with mucinous adenocarcinomas had a higher proportion of females and higher rate of combined resection than the other groups. Compared to patients with benign tumors and mucinous adenocarcinomas, patients with non‐mucinous adenocarcinomas were older and had higher rates of emergency surgery and open approach.

**Table 2:** Patient baseline characteristics by histological type (n = 230)

Variables	Total	Benign tumors (including LAMN)	Adenocarcinoma, n = 128	
			Mucinous	Non‐mucinous
	n = 230	n = 102	n = 56	n = 72
Age in years, median (range)	78 (18‐94)	65 (18‐94)	64 (36‐85)	78 (34‐89)
Gender, n (%)				
Male	93 (40.4)	41 (40.2)	17 (30.4)	35 (48.6)
Female	135 (58.7)	60 (58.8)	39 (69.6)	36 (50.0)
Unknown	2 (0.9)	1 (1.0)	0 (0.0)	1 (1.4)
Surgery, n (%)				
Elective	178 (77.4)	83 (81.3)	47 (83.9)	48 (66.6)
Emergency	47 (20.4)	17 (16.7)	8 (14.3)	22 (30.6)
Unknown	5 (2.2)	2 (2.0)	1 (1.8)	2 (2.8)
Surgical stages, n (%)				
One‐stage	190 (82.6)	96 (94.1)	42 (75.0)	52 (72.2)
Two‐stage	25 (10.9)	0 (0.0)	10 (17.8)	15 (20.8)
Others	3 (1.3)	0 (0.0)	2 (3.6)	1 (1.4)
Unknown	12 (5.2)	6 (5.9)	2 (3.6)	4 (5.6)
Surgical approach, n (%)				
Open	127 (55.2)	38 (37.2)	36 (64.3)	53 (73.6)
Laparo	98 (42.6)	62 (60.8)	20 (35.7)	16 (22.2)
Unknown	5(2.2)	2 (2.0)	0 (0.0)	3 (4.2)
Surgical procedure, n (%)				
Appendectomy	50 (21.7)	39 (38.2)	6 (10.7)	5 (7.0)
Cecal resection	8 (3.5)	8 (7.8)	0 (0.0)	0 (0.0)
Ileocecal resection	146 (63.5)	50 (49.0)	43 (76.8)	53 (73.6)
Right hemicolectomy	11 (4.8)	2 (2.0)	3 (5.4)	6 (8.3)
Others	12 (5.2)	1 (1.0)	4 (7.1)	7 (9.7)
Unknown	3 (1.3)	2 (2.0)	0 (0.0)	1 (1.4)
Lymph node dissection, n (%)				
Yes	158 (68.7)	53 (52.0)	46 (82.1)	59 (81.9)
No	69 (30.0)	49 (48.0)	9 (16.1)	11 (15.3)
Unknown	3 (1.3)	0 (0.0)	1 (1.8)	2 (2.8)
Combined resection, n (%)				
Yes	57 (24.8)	12 (11.8)	23 (41.1)	22 (30.6)
No	167 (72.6)	90 (88.2)	33 (58.9)	44 (61.1)
Unknown	6 (2.6)	0 (0.0)	0 (0.0)	6 (8.3)
Residual tumor, n (%)				
R0	177 (76.9)	93 (91.2)	37 (66.0)	47 (65.2)
R1	8 (3.5)	3 (2.9)	2 (3.6)	3 (4.2)
R2	45 (19.6)	6 (5.9)	17 (30.4)	22 (30.6)

LAMN, low‐grade appendiceal mucinous neoplasm.

### 3.2 Survival

During follow‐up, 39 patients (16.9%) died from appendiceal tumors, and there were a total of 49 (21.3%) all‐cause deaths. In two cases, death occurred ≥70 months after diagnosis, even with benign tumors. Two patients diagnosed with LAMN had tumor‐related deaths more than 5 years after surgery. In one of these cases, the patient underwent R2 resection, and was diagnosed with LAMN (T3N0M1b) with disseminated pseudomyxoma peritonei (PMP). In the other case, the patient underwent ICR with combined resection of ovary, uterus, and rectum, and was also diagnosed with LAMN (TisN0M1b) with PMP. At 14 months after surgery, this patient exhibited recurrence of pleural dissemination. Among stage 0/I mucinous adenocarcinomas, there were three cases of death. Table S1 presents three cases of stage 0/I mucinous adenocarcinomas with appendiceal tumor‐related deaths. In one of these cases (Case 1), the patient underwent ICR with combined partial resection of bladder. At 10 months after the initial surgery, this patient exhibited recurrence of peritoneal dissemination and adrenal, and died from appendiceal tumor. Three patients exhibited recurrence in two ways: hematogenous and peritoneal dissemination, but there were no recurrent cases regarding PMP.

**Figure 2:**
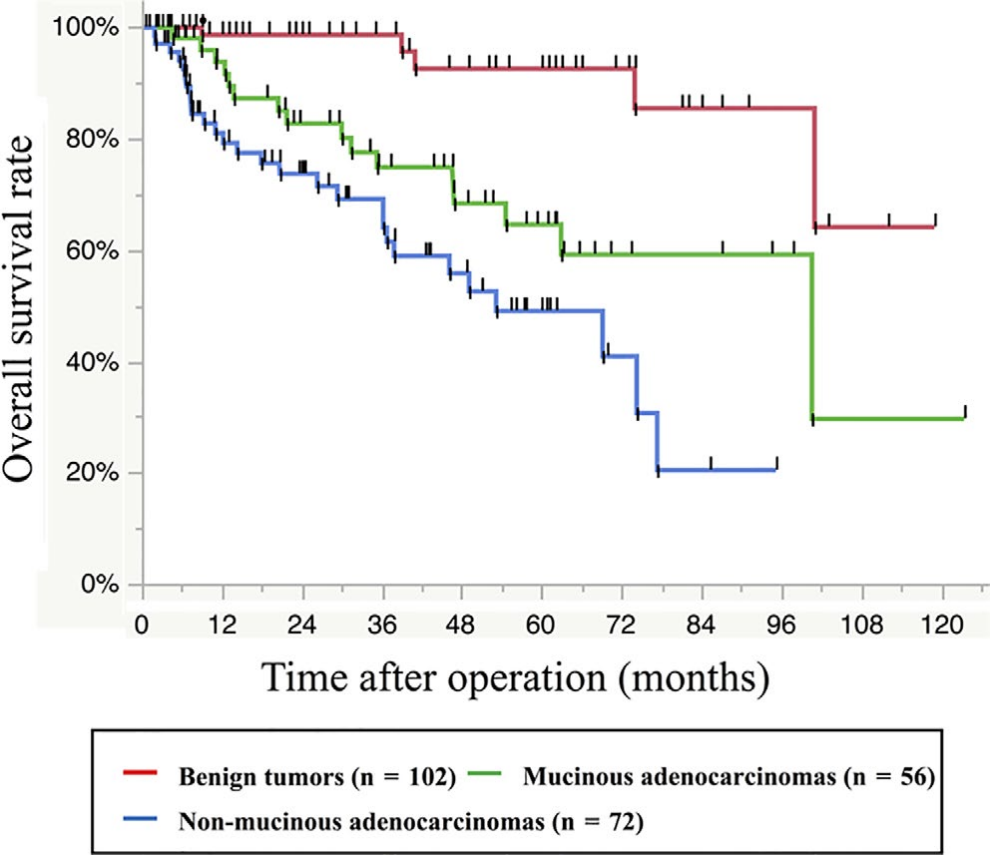
Kaplan–Meier curves of overall survival stratified by histological type

Figure 2 shows Kaplan–Meier curves of OS stratified by histological type. The 5‐year OS rates were 92.6% for benign tumors, 64.5% for mucinous adenocarcinoma, and 49.0% for non‐mucinous adenocarcinomas. OS was significantly longer for patients with benign tumors compared to those with mucinous adenocarcinomas (HR 5.09, 95% CI: 1.98‐15.6; *P *<* *0.01) and non‐mucinous adenocarcinomas (HR 9.96, 95% CI: 4.08‐30.0; *P *<* *0.01). Furthermore, patients with non‐mucinous adenocarcinomas had significantly shorter OS than those with mucinous adenocarcinomas (HR 1.95, 95% CI: 1.05‐3.75; *P *=* *0.03).

**Figure 3:**
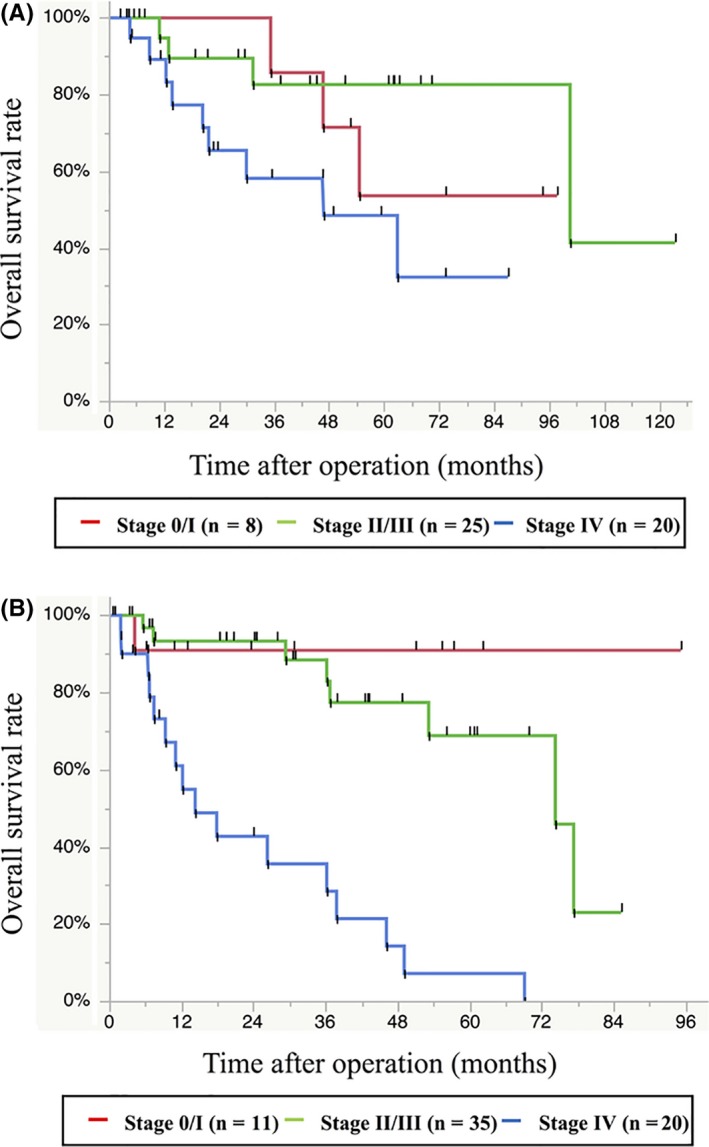
Kaplan–Meier curves of overall survival stratified by stage for (A) mucinous adenocarcinomas (n = 53), and (B) non‐mucinous adenocarcinomas (n = 66)

**Figure 4:**
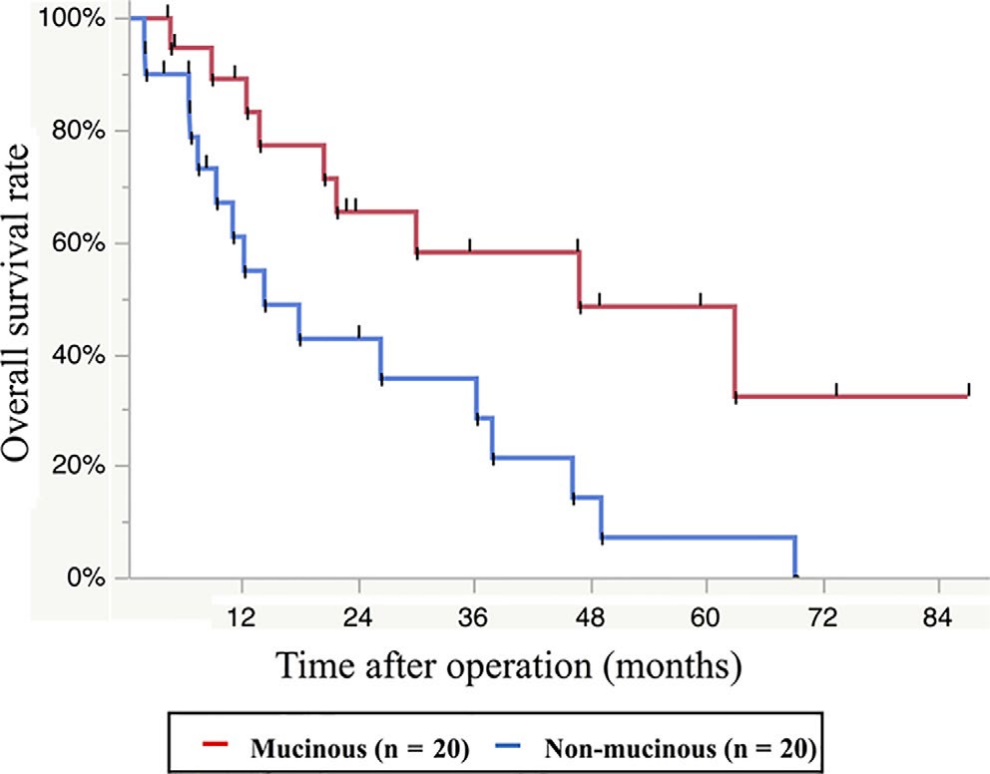
Kaplan–Meier curves of overall survival in cases of stage IV disease for mucinous adenocarcinomas (n = 20) and non‐mucinous adenocarcinomas (n = 20)

To examine the impact of staging on prognosis, we analyzed UICC stage‐stratified OS for both mucinous and non‐mucinous adenocarcinomas. Among mucinous adenocarcinomas (excluding cases with unknown stage), the overall stage distribution was 15.1% stage 0/I (n = 8), 47.2% stage II/III (n = 25), and 37.7% stage IV (n = 20). Figure 3A shows Kaplan–Meier plots of OS rates for mucinous adenocarcinomas stratified by stage. The median follow‐up was 39.1 months. The 5‐year OS rates for each stage were 53.6% for 0/I, 82.6% for II/III, and 48.4% for IV. Prognosis was poor for stage 0/I, and relatively good for stage IV, Table 3. On the other hand, among non‐mucinous adenocarcinomas (excluding cases with unknown stage), 16.7% were stage 0/I (n = 11), 53.0% stage II/III (n = 35), and 30.3% stage IV (n = 20). Figure 3B shows Kaplan–Meier plots of OS rates for non‐mucinous adenocarcinomas stratified by stage. The median follow‐up was 30.1 months. The 5‐year OS rates were 90.9% for 0/I, 68.8% for II/III, and 7.1% for IV. Higher UICC stage was associated with increased risk of death among patients with non‐mucinous adenocarcinomas (Table 3). Analysis of patients with stage IV disease revealed that OS was significantly shorter among patients with non‐mucinous adenocarcinomas compared to mucinous adenocarcinoma (HR 2.81, 95% CI: 1.25‐6.70; *P *=* *0.01; Figure 4). Regarding TNM categories, mucinous and non‐mucinous adenocarcinomas did not significantly differ in the rates of pathological T stage (*P *=* *0.12) or N stage (*P *=* *0.14). Table S2 shows the 5‐year OS rates stratified by TNM categories for mucinous and non‐mucinous adenocarcinomas. Higher TNM grade predicted an increased risk of death in cases of non‐mucinous adenocarcinomas, but not mucinous adenocarcinoma (Table S2).

**Table 3:** Survival outcome stratified by UICC stage for mucinous or non‐mucinous adenocarcinomas

Variables	n (%)	5‐y OS rate (%)	HR (95% CI)	*P* value
Mucinous adenocarcinoma (excluding cases with unknown stage)	53			
0/I	8 (15.1)	53.6	1.00 (reference)	–
II/III	25 (47.2)	82.6	0.48 (0.08‐2.60)	0.37
IV	20 (37.7)	48.4	1.97 (0.58‐8.99)	0.28
Non‐mucinous adenocarcinoma (excluding cases with unknown stage)	66			
0/I	11 (16.7)	90.9	1.00 (reference)	–
II/III	35 (53.0)	68.8	2.88 (0.52‐53.6)	0.25
IV	20 (30.3)	7.1	20.9 (4.01‐389.2)	<0.01

CI, confidence interval; HR, hazard ratio; OS, overall survival; UICC, Union for International Cancer Control.

The 5‐year OS rates for mucinous adenocarcinomas were 50.0% (well differentiated), 80.0% (moderately differentiated), 50.0% (poorly differentiated), and 63.8% (unknown differentiation). For non‐mucinous adenocarcinomas, the 5‐year OS rates were 91.6% (well differentiated), 39.2% (moderately differentiated), 28.1% (poorly differentiated), 37.5% (signet‐ring cell carcinoma), and 0.0% (unknown differentiation) (Table S3). We constructed Kaplan–Meier plots of OS rates for non‐mucinous adenocarcinomas stratified by differentiation grade (Figure S1). Unknown differentiation was significantly more common for mucinous adenocarcinomas (67.8%) than for non‐mucinous adenocarcinomas (6.9%). Among non‐mucinous adenocarcinomas, well‐differentiated cases had significantly better outcomes than moderately differentiated (*P *=* *0.02) and poorly differentiated (*P *=* *0.08) cases. This association was not found among mucinous adenocarcinomas (Table S3).

## 4 DISCUSSION

The results of our present multicenter retrospective clinical study highlight the interplay between histological types and stages among patients with appendiceal neoplasms, and show how these factors impact prognosis between mucinous and non‐mucinous adenocarcinomas. In two cases, death occurred more than 5 years after surgery, even with benign tumors. These findings suggest that these cases should be considered mucinous adenocarcinoma due to the diagnosis of LAMN. Our results also revealed a better prognosis in patients with mucinous adenocarcinomas compared to non‐mucinous adenocarcinomas in this setting. Furthermore, higher UICC stage was associated with increased risk of death among patients with non‐mucinous adenocarcinomas, but not with mucinous adenocarcinomas.

There is presently scarce evidence regarding the appropriate management and surveillance of patients with appendiceal mucinous neoplasms. LAMN is by far the most frequent source of PMP, and can develop intraperitoneal recurrence many years after the initial presentation.^7^, ^15^, ^16^, ^17^ Therefore, patients with LAMN should routinely be offered follow‐up to monitor for subsequent PMP development.^18^ In fact, our data showed that two patients with LAMN died due to their disease more than 5 years after their initial surgery. A reasonable follow‐up schedule for LAMN would include abdominopelvic computerized tomography (CT) scans with tumor markers—including carcinoembryonic antigen (CEA), cancer antigen 125 (CA125), and carbohydrate antigen 19‐9 (CA19‐9)—at 1 year, and then annually for several years, with decreasing frequency over time. If spread of cells or mucin beyond the appendix is not detected, 5‐10 years of follow‐up is probably adequate, but otherwise it should be longer.^19^

Unfortunately, our present data cannot clarify the relationship between appendiceal mucinous adenocarcinomas and PMP. PMP is a clinical syndrome in which a mucinous neoplasm grows within the peritoneal cavity causing mucinous ascites and peritoneal implants.^19^ It is uncommon, with an incidence of approximately 0.2 per 100 000 per year.^17^ Actually, no recurrence cases due to PMP were revealed among appendiceal mucinous adenocarcinomas in this study. However, in the present setting we analyzed a cohort of patients who had been histologically diagnosed with appendiceal tumors, and no patients with carcinomas of unknown primary are included. Furthermore, the diagnostic criteria of PMP and appendiceal mucinous adenocarcinomas have not yet become sufficiently understood in general among pathologists, despite accurate diagnosis of both appendiceal mucinous adenocarcinomas and PMP being so important.^19^ Thus, it is difficult to clarify the incidence rate of death due to PMP among appendiceal mucinous adenocarcinomas.

Consistent with previous reports,^13^, ^20^ we identified a number of differences between mucinous and non‐mucinous adenocarcinomas. Female sex and disease stage IV were more common for mucinous adenocarcinomas. On the other hand, stage III disease was less common for mucinous adenocarcinomas than non‐mucinous adenocarcinomas, suggesting that mucinous adenocarcinomas showed preferential peritoneal spread rather than lymphatic spread. In a recent large retrospective study of mucinous and non‐mucinous colorectal adenocarcinomas, mucinous adenocarcinomas were more commonly found at a more advanced stage, and were predominantly located at the right side of the colon.^21^ They also reported that patients with mucinous adenocarcinomas were younger than those with non‐mucinous adenocarcinomas,^21^ which was supported by our present findings. However, we did not find significantly different rates of pathological T stage between mucinous and non‐mucinous adenocarcinomas. Moreover, in contrast to prior results, we found that patients with non‐mucinous adenocarcinomas had more advanced stages than those with mucinous adenocarcinomas. The presently available data indicate potential differences between appendiceal mucinous adenocarcinomas and colorectal mucinous adenocarcinomas. Our present analysis was limited by sample size, and thus further studies are required to resolve this issue. On the other hand, the oncologic behavior of appendiceal non‐mucinous adenocarcinomas was similar to that reported for colorectal non‐mucinous adenocarcinomas.

Interestingly, prior retrospective series have demonstrated both better^22^, ^23^, ^24^ and worse^25^, ^26^ prognoses among patients with mucinous compared to non‐mucinous histological subtypes. An analysis of the Surveillance, Epidemiology and End‐Results (SEER) registry from 1973 to 1988 showed that all‐cause survival did not differ between patients with mucinous versus non‐mucinous adenocarcinomas.^13^ A recent analysis of SEER data reported that the 5‐year OS rates were 53.6% for patients with mucinous adenocarcinoma, and 46% for non‐mucinous adenocarcinoma.^27^ In our present study, the 5‐year OS rates were 64.5% for mucinous adenocarcinomas, and 49.0% for non‐mucinous adenocarcinomas, and patients with non‐mucinous adenocarcinomas had significantly shorter OS than those with mucinous adenocarcinomas. Thus, our findings revealed a better prognosis in patients with mucinous adenocarcinomas compared to non‐mucinous adenocarcinomas, although our analysis was limited by sample size. When our analysis was limited to patients with stage IV disease, mucinous adenocarcinoma was associated with better prognosis compared to non‐mucinous adenocarcinomas, similar to previously published data.^28^

Another notable finding from our study was that UICC staging was not associated with prognosis in mucinous adenocarcinoma. Interestingly, higher UICC stage was associated with increased risk of death among patients with non‐mucinous adenocarcinomas, but not among patients with mucinous adenocarcinoma. However, previous reports show stage‐dependent survival in patients with mucinous adenocarcinoma.^27^, ^28^ Our findings are likely influenced by the small number of patients, as well as the high rate of patients lacking lymph node dissection. Moreover, many cases with benign or uncertain malignant potential may have been diagnosed as LAMN, but these cases were not considered mucinous adenocarcinomas. Our results suggest a need to re‐evaluate pathological histology in benign tumors. Finally, it is possible that the stage was underestimated at the time of surgery for mucinous adenocarcinomas. Intraoperative findings, such as micro‐sowing, are important not only for selection of surgical procedure but also for identifying patients with poor prognosis.

The present study has both strengths and limitations. One of its strengths is that the dataset was based on the medical records from each institution participating in a multicenter collaborative research group with the aim of investigating the histological types and stages of appendiceal neoplasms. This enabled us to evaluate precise data relating to mucinous adenocarcinomas and non‐mucinous adenocarcinomas, which may be difficult to identify in a larger cohort. One limitation of our study is its retrospective design. It is unknown whether the study included patients with mixed‐type adenomas. Additionally, the number of patients was small and the follow‐up period was not matured. Therefore, our data may not be sufficient to draw definitive conclusions, compared to previous studies with larger cohorts.^27^, ^28^, ^29^ It should also be noted that patients with missing survival data were excluded from survival analysis, which could potentially introduce bias.

Another potential limitation of this study is the high percentage of mucinous adenocarcinomas with unknown differentiation, which was significantly higher than the percentage of non‐mucinous adenocarcinomas with unknown differentiation. Moreover, the 67.8% rate of unknown differentiation among mucinous adenocarcinomas was significantly higher than the rates of 32% and 35% reported in the National Cancer Data Base and SEER databases, respectively.^27^, ^28^, ^29^ Thus, the evidence from our present study could not support the current AJCC 8th and PSOGI classification, in which appendiceal mucinous adenocarcinomas were classified into three tiers: well (G1), moderately (G2), and poorly differentiated (G3). In fact, it is difficult to obtain reliable data for appendiceal mucinous adenocarcinomas due to the inconsistent definitions used in the literature. Additionally, despite advances of knowledge in the field of gastroenterological surgery, appendiceal carcinomas are not mentioned in the Japanese Society for Cancer of the Colon and Rectum (JSCCR) guidelines for colorectal cancer treatment.^30^, ^31^ However, large retrospective cohort studies have demonstrated that differentiation grade is important for predicting survival outcomes in patients with mucinous adenocarcinoma, and report that histological grade has a greater prognostic implication for mucinous adenocarcinomas compared to non‐mucinous adenocarcinomas.^28^, ^29^ These findings support the unique biological behavior of appendiceal mucinous adenocarcinomas. It would be interesting to investigate how knowledge of the differentiation grade of the cases with unknown differentiation in our present study might have affected the outcomes of mucinous adenocarcinomas. The 2018 Japanese Classification of Colorectal Carcinoma^32^ mentions the differentiation grade of mucinous carcinomas, supporting the possibility of future prospective studies evaluating detailed data, including the differentiation grade of appendiceal mucinous neoplasms.

## 5 CONCLUSION

Here we found that mucinous adenocarcinomas are associated with better prognosis than non‐mucinous adenocarcinomas. In the present setting, UICC staging was associated with prognosis among non‐mucinous adenocarcinomas, but not mucinous adenocarcinoma. Analysis of stage IV disease revealed better prognosis in patients with mucinous adenocarcinoma compared to non‐mucinous adenocarcinomas. Further studies are needed to confirm these findings.

## DISCLOSURE

Conflict of interest: the authors have no conflicts of interest to declare.
