# Supplementary figures and images for: Survival outcomes of appendiceal mucinous neoplasms by histological type and stage: Analysis of 266 cases in a multicenter collaborative retrospective clinical study
Source: Ann Gastroenterol Surg. 2019 Feb 25;3(3):291–300. doi: 10.1002/ags3.12241 (PMC6524118; doi:10.1002/ags3.12241)

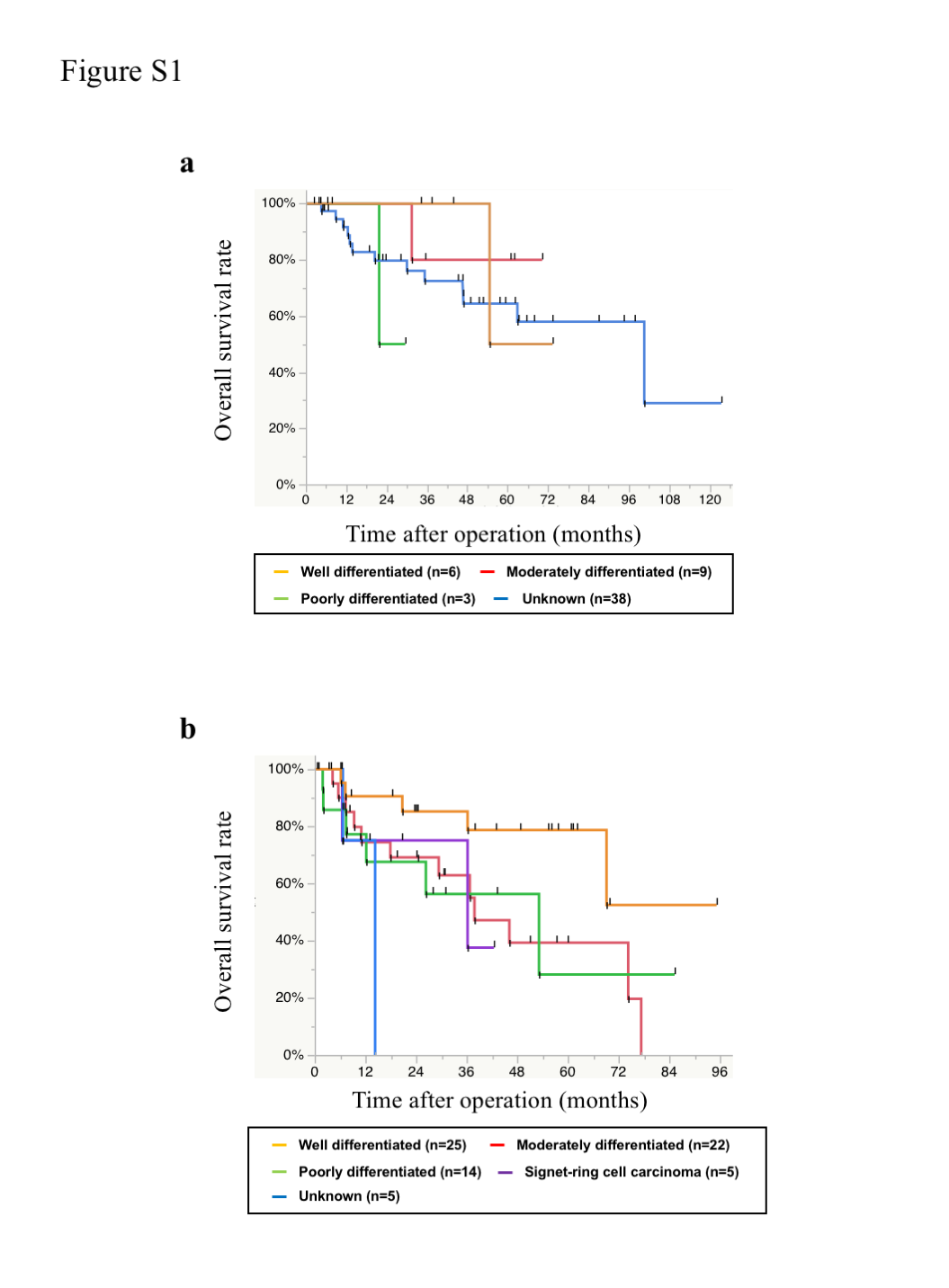

Supplement: Supplementary file 1 [file AGS3-3-291-s001.tiff]
