# Supplementary material for: Survival outcomes of appendiceal mucinous neoplasms by histological type and stage: Analysis of 266 cases in a multicenter collaborative retrospective clinical study
Source: Ann Gastroenterol Surg. 2019 Feb 25;3(3):291–300. doi: 10.1002/ags3.12241 (PMC6524118; doi:10.1002/ags3.12241)
Supplement: Supplementary file 2 [file AGS3-3-291-s002.docx]

**Supplementary TABLE 1** Three cases of stage 0/I mucious adenocarcinomas with appendiceal tumors-related death

| **Case** | **Age**  **Sex** | **Surgical procedure** | **Lymph**  **node dissection** | **Differentiation grade** | **T**  **N**  **M** | **R** | **Recurrence site**  **Recurrence-free survival**  **(months)** | **Survival after surgery**  **(months)** |
| --- | --- | --- | --- | --- | --- | --- | --- | --- |
| 1 | 81  M | Ileocecal resection | Yes | Unknown | Tis  N0  M0 | R0 | Peritoneal dissemination  Adrenal  10 | 35.3 |
| 2 | 85  M | Ileocecal resection | Yes | Well differentiated  (G1) | T1  N0  M0 | R0 | Liver  48 | 54.6 |
| 3 | 82  F | Ileocecal resection | Yes | Unknown | T2  N0  M0 | R0 | Ovary  27 | 46.7 |

R, Residual tumor.

**Supplementary TABLE 2** Survival outcome stratified by TNM categories for mucinous and non-mucinous adenocarcinomas

| **Variables** | **n (%)** | **5-y OS rate, %** | **HR (95% CI)** | ***P* value** |
| --- | --- | --- | --- | --- |
| **Mucinous adenocarcinoma** | 56 |  |  |  |
| **T category** |  |  |  |  |
| Tis/T1/T2 | 9 (16.1) | 53.6 | 1.00 (reference) | − |
| T3/T4 | 39 (69.6) | 75.6 | 0.82 (0.23–3.86) | 0.79 |
| TX | 8 (14.3) | 50.0 | 1.97 (0.48–9.61) | 0.32 |
| **N category** |  |  |  |  |
| N0 | 35 (62.5) | 65.9 | 1.00 (reference) | − |
| N1/N2 | 12 (21.4) | 79.6 | 1.28 (0.27–4.58) | 0.72 |
| NX | 9 (16.1) | 55.6 | 3.15 (0.94–9.59) | 0.06 |
| **M category** |  |  |  |  |
| M0 | 36 (64.3) | 74.1 | 1.00 (reference) | − |
| M1 | 20 (35.7) | 48.4 | 3.45 (1.23–10/3) | 0.02 |
|  |  |  |  |  |
| **Non-mucinous adenocarcinoma** | 72 |  |  |  |
| **T category** |  |  |  |  |
| Tis/T1/T2 | 14 (19.4) | 80.8 | 1.00 (reference) | − |
| T3/T4 | 55 (76.4) | 45.4 | 3.16 (0.93–19.7) | 0.06 |
| TX | 3 (4.2) | 0.0 | 14.7 (2.35–116.4) | <0.01 |
| **N category** |  |  |  |  |
| N0 | 36 (50.0) | 69.2 | 1.00 (reference) | − |
| N1/N2 | 27 (37.5) | 20.2 | 2.77 (1.18–6.63) | 0.02 |
| NX | 9 (12.5) | 18.8 | 5.29 (1.75–14.8) | <0.01 |
| **M category** |  |  |  |  |
| M0 | 47 (65.3) | 74.3 | 1.00 (reference) | − |
| M1 | 20 (27.8) | 7.1 | 8.45 (3.56–22.2) | <0.01 |
| MX | 5 (6.9) | 30.0 | 6.27 (1.34–22.8) | <0.01 |

OS, overall survival; HR, hazard ratio; CI, confidence interval.

**Supplementary TABLE 3** Survival outcome stratified by differentiation grade for mucinous and non-mucinous adenocarcinomas

| **Variables** | **n (%)** | **5-y OS rate, %** | **HR (95% CI)** | ***P* value** |
| --- | --- | --- | --- | --- |
| **Mucinous adenocarcinoma** |  |  |  |  |
| **Differentiation grade** | 56 |  |  |  |
| Well differentiated (G1) | 6 (10.7) | 50.0 | 1.00 (reference) | − |
| Moderately differentiated (G2) | 9 (16.1) | 80.0 | 0.90 (0.03–22.8) | 0.94 |
| Poorly differentiated (G3) | 3 (5.4) | 50.0 | 4.93 (0.18–129.3) | 0.28 |
| Unknown | 38 (67.8) | 63.8 | 1.94 (0.38–35.5) | 0.48 |
|  |  |  |  |  |
| **Non-mucinous adenocarcinoma** |  |  |  |  |
| **Differentiation grade**  **(excluding cases with pap)** | 71 |  |  |  |
| Well differentiated | 25 (34.7) | 91.6 | 1.00 (reference) | − |
| Moderately differentiated | 22 (30.6) | 39.2 | 3.06 (1.07–8.75) | 0.02 |
| Poorly differentiated | 14 (19.4) | 28.1 | 2.87 (0.86–9.54) | 0.08 |
| Signet-cell carcinoma | 5 (6.9) | 37.5 | 3.25 (0.61–17.0) | 0.20 |
| Unknown | 5 (6.9) | 0.0 | 5.65 (1.01–31.3) | 0.08 |

OS, overall survival; HR, hazard ratio; CI, confidence interval; pap, papillary adenocarcinoma.
