# Supplementary material for: Survival outcomes of appendiceal mucinous neoplasms by histological type and stage: Analysis of 266 cases in a multicenter collaborative retrospective clinical study
Source: Ann Gastroenterol Surg. 2019 Feb 25;3(3):291–300. doi: 10.1002/ags3.12241 (PMC6524118; doi:10.1002/ags3.12241)
Supplement: Supplementary file 3 [file AGS3-3-291-s003.docx]

**SUPPLEMENTARY INFORMATION**

1. **Title: Survival outcomes of appendiceal mucinous neoplasms by histological type and stage: Analysis of 266 cases in a multicenter collaborative retrospective clinical study**
2. Authors:
   Toshinori Sueda*, MD, PhD1, 2, Kohei Murata*, MD, PhD1, 3, Takashi Takeda, MD1, 4, Yoshinori Kagawa, MD, PhD1, 3, Junichi Hasegawa, MD, PhD1, 2, Takamichi Komori, MD, PhD1, 5, Shingo Noura, MD, PhD 1, 6, Kimimasa Ikeda, MD, PhD 1, 7, Masaki Tsujie, MD, PhD 1, 8, Masayuki Ohue, MD, PhD 1, 9, Hirofumi Ota, MD, PhD 1, 10, Masakazu Ikenaga, MD, PhD 1, 11, Taishi Hata, MD, PhD1, 4, Chu Matsuda, MD, PhD1, 4, Tsunekazu Mizushima, MD, PhD1, 4, 12, Hirofumi Yamamoto, MD, PhD1, 4, 13, Mitsugu Sekimoto, MD, PhD1, 14, Riichiro Nezu, MD, PhD, 1,15, Masaki Mori, MD, PhD1, 16 and Yuichiro Doki, MD, PhD1, 4

*These authors equally contributed to this study.

1 Clinical Study Group of Osaka University (CSGO), Colorectal Group

2 Department of Surgery, Osaka Rosai Hospital, Sakai, Japan

3 Department of Surgery, Kansai Rosai Hospital, Amagasaki, Japan

4 Department of Gastroenterological Surgery, Osaka University Graduate School of

Medicine, Suita, Japan

5 Department of Surgery, Osaka General Medical Center, Osaka, Japan

6 Department of Surgery, Toyonaka Municipal Hospital, Toyonaka, Japan

7 Department of Surgery, Minoh City Hospital, Minoh, Japan

8 Department of Surgery, Sakai City Medical Center, Sakai, Japan

9 Department of Gastroenterological Surgery, Osaka International Cancer Institute,

Osaka, Japan

　　10 Department of Surgery, Ikeda City Hospital, Ikeda, Japan

11 Department of Gastroenterological Surgery, Higashiosaka City Medical Center,
 Higashiosaka, Japan

12 Department of Therapeutics for Inflammatory Bowel Diseases,

Graduate School of Medicine, Osaka University, Suita, Japan

13 Division of Health Sciences, Graduate School of Medicine, Osaka University,

Suita, Japan

14 Department of Surgery, National Hospital Organization Osaka National

Hospital, Osaka, Japan

15 Department of Surgery, Nishinomiya Municipal Central Hospital, Nishinomiya,
 Japan

16 Department of Surgery and Science, Graduate School of Medical Sciences,

Kyushu University, Fukuoka, Japan

## Correspondence:

Kohei Murata, MD, PhD

Department of Surgery, Kansai Rosai Hospital, Amagasaki, Japan

3-1-69 Inabaso, Amagasaki, Osaka, 660-8511 Japan

TEL: +81-6-6416-1221

E-mail: kmuratajp@yahoo.co.jp

**FIGURE LEGENDS**

**FIGURE S1** Kaplan-Meier curves of overall survival stratified by differentiation grade for (a) mucinous adenocarcinomas (n = 56), and (b) non-mucinous adenocarcinomas (n = 71).
